# Is vitamin D deficiency influenced by obesity during the first 5 years of life? A cross‐sectional multicenter study

**DOI:** 10.1002/fsn3.3145

**Published:** 2022-11-17

**Authors:** Yan Zhao, Rui Qin, Hong Hong, Heyu Lv, Kan Ye, Yarong Wei, Wen Zheng, Hongxia Qi, Yufei Ni, Li Zhang, Guoqiang Yang, Guoqin Liu, Aiping Wu

**Affiliations:** ^1^ Department of Clinical Nutrition Jiangsu Province Hospital, The First Affiliated Hospital of Nanjing Medical University Nanjing China; ^2^ Department of Child Health Care, Jiangsu Women and Children Health Hospital, Women and Child Branch Hospital of Jiangsu Province Hospital The First Affiliated Hospital of Nanjing Medical University Nanjing China; ^3^ Department of Child Health Care Drum Tower Maternity and Child Health Care Institute Nanjing China; ^4^ Department of Child Health Care Jiangning Maternity and Child Health Care Institute Nanjing China; ^5^ Department of Child Health Care Suzhou Municipal Hospital Suzhou China; ^6^ Department of Child Health Care Wuxi Maternity and Child Health Care Hospital Wuxi China; ^7^ Department of Child Health Care Yancheng Maternity and Child Health Care Institute Yancheng China; ^8^ Department of Child Health Care Xuzhou Children's Hospital Xuzhou China; ^9^ Department of Child Health Care Nantong Maternity and Child Health Care Hospital Nantong China; ^10^ Department of Child Health Care Huai'an Maternity and Child Health Care Hospital Huai'an China; ^11^ Department of Child Health Care Kunshan Maternity and Child Health Care Institute Kunshan China; ^12^ Department of Child Health Care Dafeng Maternity and Child Health Care Hospital Dafeng China; ^13^ Department of Child Health Care Xinghua Maternity and Child Health Care Hospital Xinghua China

**Keywords:** early life, body mass index, children, infant, obesity, vitamin D

## Abstract

Evidence on the association of 25‐hydroxyvitamin D (25[OH]D) and obesity during the first 5 years of life is limited in China. The objective of this study was to examine the associations between weight, weight for age *z* score (ZWAZ), weight for length/height *z* score (ZWHZ), and body mass index for age *z* score (ZBMI) and 25(OH)D. This was a large population‐based cross‐sectional multicenter study in which the children aged 0–5 years were recruited from 12 children's healthcare centers by a stratified cluster random‐sampling method in 10 cities of the Jiangsu province, China. The 25(OH)D concentration was determined by ELISA. A total of 5289 children were investigated. For 0–71 months children with obesity and nonobesity, the prevalence of vitamin D deficiency was 36.0% and 29.8%, and the 25(OH)D level was 59.8 and 64.0 nmol/L, respectively, and there were all significant difference. Compared with children with nonobesity, children with obesity had higher risk of vitamin D deficiency (OR [95% CI]: 1.33 [1.02, 1.72], *p* < .05), and had lower 25(OH)D level (β = −3.84, 95% CI = −7.58, −0.09, *p* < .05). The results for children aged 24–71 months were similar to those for children aged 0–71 months. However, no significant difference was observed in children aged 0–23 months. Vitamin D deficiency was observed in children with greater adiposity during the first 5 years of life. However, the results mainly came from those in the age group of 2 to 5 years instead of the first 2 years in their lives.

## Graphical abstract

Vitamin D deficiency was observed in children with greater adiposity during the first 5 years of life. However, the results mainly came from those in the age of 2 to 5 years old instead of the first 2 years in their lives.

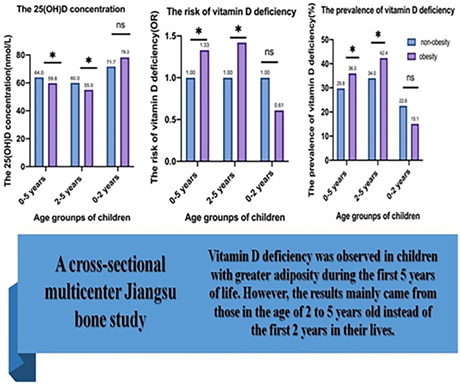

## 1 INTRODUCTION

Vitamin D deficiency is highly prevalent and has become a common observation for pediatric population. Our results showed that the median serum 25‐hydroxyvitamin D (25[OH]D) concentration was 62.9 nmol/L and 28.9% of the children had vitamin D deficiency among children in the Jiangsu province, China (Fu et al., 2016). Vitamin D deficiency has been a growing concern in recent years, despite constant efforts to lower its prevalence, it remains a widespread issue not only throughout China, but also worldwide. Of preschoolers, 58% (all 5–6 years of age) from southern Croatia had 25(OH)D levels of <50 nmol/L (Karin et al., 2018). Hypovitaminosis D, defined as serum 25(OH)D concentration <50 nmol/L, was found in 91.1% of the healthy children aged 12–60 months in rural Nepal (Avagyan et al., 2016). Prevalence of vitamin D deficiency was 77% among Jordanian healthy infants (Kassab et al., 2016).

The overall obesity prevalence among Chinese children under 7 years of age from 1986 to 2016 was from 0.91% to 4.2%. In the last 30 years, obesity prevalence of Chinese children under 7 years of age was highly increased by 4.62 times (“Capital Institute of Pediatrics, The Coordinating Study Group of Nine Cities on the Physical Growth and Development of Children. A national epidemiological survey on obesity of children under seven years of age in nine cities of China in 2016,” 2018). Both vitamin D deficiency and obesity are common conditions in the pediatric population. In addition, the role of vitamin D in adipose tissue includes the regulation of lipolysis and adipogenesis, adipocyte differentiation, proliferation and apoptosis, cytokine release, adipose tissue inflammation, oxidative stress, etc. (Abbas, 2017).

Preobesity and obesity in children aged 7–15 years are inversely associated with vitamin D status (Jaksic et al., 2019). The prevalence of vitamin D deficiency (serum 25[OH]D <50 nmol/L) was higher in obese children aged 9–13 years compared to their over‐ and normal weight counterparts (60.5% vs. 51.6% and 51%, *p* = .017; Moschonis et al., 2018). Obesity is associated with vitamin D deficiency in Danish children and adolescents aged 6–18 years (Plesner et al., 2018). 25‐Hydroxvitamin D concentrations are not lower in 6‐ to 13‐year‐old children with obesity (Reinehr et al., 2018). Total vitamin D was negatively correlated with BMI SDS in children (age: 12.8 ± 0.2 years; Corica et al., 2019). The interests of previous studies have mainly evaluated vitamin D status and obesity in school children and adolescents (Corica et al., 2019; Jaksic et al., 2019; Moschonis et al., 2018; Plesner et al., 2018; Reinehr et al., 2018), but data worldwide focusing on the first 5 years of life are lacking, to the best of our knowledge. The aim of this study was to determine possible association between obesity and 25(OH)D in Chinese children during the first 5 years of life.

## 2 METHODS

### 2.1 Participants and study design

**FIGURE 1:**
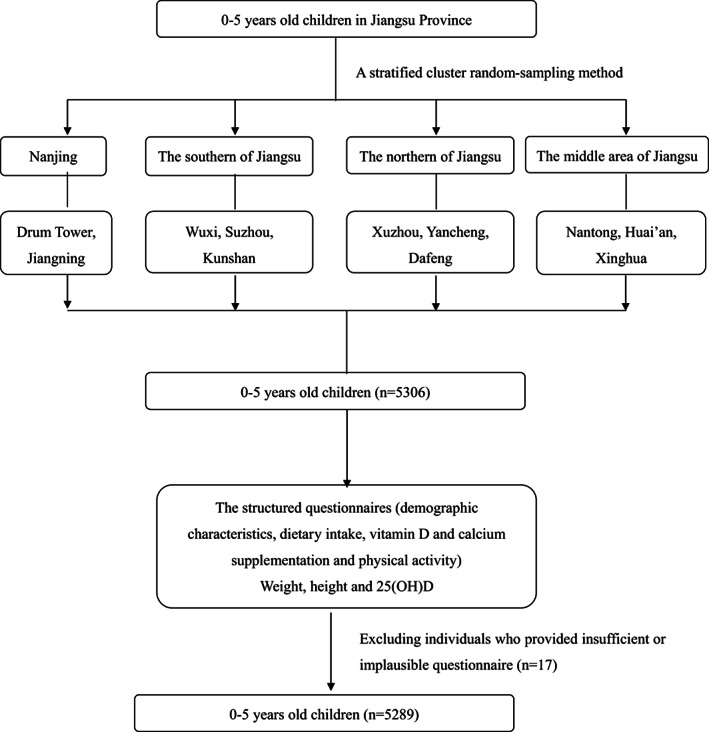
Flowchart of children through the study.

In this study, children were recruited from the Jiangsu province between April 2014 and March 2015. This was a large population‐based cross‐sectional multicenter study in which the children aged 0–5 years were recruited from 12 children's healthcare centers in 10 cities of the Jiangsu province, China. The sampling method and inclusion/exclusion criteria were reported in a previous study (Zhao et al., 2020). In brief, Jiangsu province is divided into four districts including Nanjing (between 31°14′ and 32°36′ north latitude), the southern of Jiangsu province (between 31°06′ and 32°2′ north latitude), the northern of Jiangsu province (between 32°34′ and 34°28′ north latitude), and the middle area of Jiangsu province (between 31°41′ and 34°06′ north latitude). From each district, 3–4 sampling children's healthcare centers were randomly drawn. The children with metabolic bone disease or abnormal PTH level were excluded. The parents of the children were face to face interviewed using structured questionnaires, and birth weight, weight, height, and serum 25(OH)D concentration for the children were measured. The study profile is presented in Figure 1.

### 2.2 Assessment of demographic and lifestyle factors

The structured questionnaire included the information on the children's and mother's demographic characteristics including gender, age, number of pregnancies, parity, gestational age, delivery mode, birth weight, region of residence, season of survey, and location in Jiangsu province. The lifestyle factors included feeding patterns for infants from birth to 6 months (breast milk, formula milk, or mixed), the dietary intake, vitamin D and calcium supplementation, and physical activity.

### 2.3 Assessments of dietary intake and physical activity

The parent or caregiver was asked to indicate his or her child's frequency and portion size (grams per times) of each food or food group in the validated self‐administered food frequency questionnaire (FFQ) consumed in the last 1 month prior to the survey. The FFQ was modified on the basis of a previous questionnaire (Lu et al., 2016). Food intake in grams per day was calculated as the reported frequency and portion size. Based on culinary usage or nutrient profiles, we further categorized each food items into 14 food groups as follows: rice and rice products, wheat and wheat products, other cereal products, tubers, starches and starch products, milk and milk products (not including human milk), meat or poultry products, fish and shrimp, eggs, fruits or fruit juices, vegetables, soups, legumes, and oils.

The parent or caregiver was also asked about the children's physical activity levels using a validated self‐reported questionnaire, type, and duration of physical activity, especially the time of sleeping and outdoor activity. The questionnaire was modified on the basis of a previous questionnaire (Lee et al., 2017). The physical activity levels were measured as typical hours of daily activity (h/day).

### 2.4 Anthropometry

Trained research assistants used standard anthropometric methods and measured weight and length/height for children. The shoes, underwear, and nappy were removed. Weight and length (at 0 and 36 months) were measured using a horizontal stadiometer (22WB‐A, China). Weight and height (at 37 and 71 months) were measured using a horizontal stadiometer (SZ‐200, China). BMI was computed as weight in kilograms divided by the square of the height in meters (“WHO Multicentre Growth Reference Study Group. WHO child growth standards: methods and development: length/height‐for‐age, weight‐for‐age, weight‐for‐length, weight‐for‐height and body mass index‐for‐age. Geneva, Switzerland: World Health Organization, 2006”; Zhao et al., 2020).

### 2.5 Vitamin D measurement

Blood samples in boxes were collected according to the time of collection. A biochemical analysis was performed within 24 h by trained technicians and serum was stored at −80°C for future analysis. Serum 25(OH)D concentration was measured by the enzyme‐linked immunosorbent assay (ELISA) kit OCTEIA 25‐Hydroxy vitamin D (Immunodiagnostic Systems, Boldon, United Kingdom) according to the manufacturer's instructions. More than 10% results were selected to repeat for quality control at random. The coefficients of variation (CV) for the 25(OH)D assay determined at assay verification were as follows: interassay CV, 8.7% at a concentration of 49.5 nmol/L and 3.6% at 103 nmol/L (intraassay CV, 2.8% and 1.3%, respectively), and the reported analytic sensitivity of the immunoassay was 6.8–380 nmol/L. The condition of blood sampling is random. The blood sample is kept in dark place. This kit has obtained the US Centers for Disease Control and Prevention (CDC) Vitamin D Standardization Certification (VDSP; Sempos et al., 2017).

### 2.6 Definition of terms

The weight for age *z* score (ZWAZ), weight for length/height *z* score (ZWHZ), and BMI for age *z* score (ZBMI) were calculated based on the World Health Organization Standard (“WHO Multicentre Growth Reference Study Group. WHO child growth standards: methods and development: length/height‐for‐age, weight‐for‐age, weight‐for‐length, weight‐for‐height and body mass index‐for‐age. Geneva, Switzerland: World Health Organization, 2006”). In children, obesity was defined as a ZBMI at or above the 95th percentile; nonobesity was defined as a ZBMI lower than the 95th percentile (Ogden et al., 2014). According to the Endocrine Society's clinical practice guidelines, vitamin D status was defined as follows: vitamin D deficiency (25[OH]D <50 nmol/L; Holick et al., 2011).

### 2.7 Statistical analysis

The basic characteristics in children with vitamin D deficiency and obesity were described as number (percentage). The basic characteristics in children with 25(OH)D level were described as median P50 (P25–P75). Mann–Whitney *U* nonparametric test or *K* independent samples median nonparametric test was used to describe median differences by continuous variables and chi‐square test was used to examine differences in categorical variables. Because the first 1000 days of life, that is, the time from mother's conception to child's 2 years of age, can significantly affect the health of children (0–71 months old is divided into two stages: 0–23 months old and 24–71 months old). The relation between weight, ZWAZ, ZWHZ, ZBMI, and 25(OH)D levels was analyzed using generalized linear model regression in the additive model. The associations between obesity with vitamin D deficiency and 25(OH)D concentrations were, respectively, evaluated using binary logistic regression and generalized linear model regression in the additive model. The additive model included the following confounders: Model 1 included sex, number of pregnancies, parity, birth weight, region of residence, season, location in Jiangsu province; Model 2 included the variables in Model 1 plus vitamin D supplementation for infants from birth to 6 months, the initial time of vitamin D supplementation after birth, vitamin D and calcium supplementation for children in the last 3 months; Model 3 included the variables in Model 1 plus daily intake of milk, meat, and egg for children; Model 4 included the variables in Model 1 plus time of sleeping and outdoor activity every day for children. All statistical analyses were conducted using IBM's SPSS software (version 25.0; SPSS, Chicago, IL), and *p* < .05 in a two‐sided test was interpreted as statistically significant.

### 2.8 Ethical consideration

The study protocol was approved by the institutional review board of the First Affiliated Hospital of Nanjing Medical University. The approval number is removed for blind peer review.

## 3 RESULTS

Table 1 illustrates the basic characteristics of vitamin D deficiency, 25(OH)D levels, and obesity during the first 5 years of life. A total of 5289 children (2786 boys and 2503 girls) were investigated. The percentage of vitamin D deficiency in children aged 0–23 months, 24–71 months, and 0–71 months in Jiangsu province were 22.2%, 34.4%, and 30.1%, respectively. The percentage of obesity in children aged 0–23 months, 24–71 months, and 0–71 months in Jiangsu province were 4.1%, 5.5%, and 5.0%, respectively. A higher prevalence of obesity was observed in basic characteristics including boys, older children, cesarean delivery, macrosomia, in winter, no vitamin D supplementation from birth to 6 months, the initial time of food supplementation ≤4 months, daily intake of meat >150.0 g, and time of sleeping <10 h every day. A higher prevalence of vitamin D deficiency or lower 25(OH)D concentration was observed in basic characteristics including older children, girls, first pregnancy, first parity, in spring, in the southern of Jiangsu, macrosomia, residence in urban, daily intake of milk <250 ml, daily intake of meat >150.0 g, daily intake of egg intake <60.0 g, no vitamin D supplementation from birth to 6 months, the initial time of vitamin D supplementation after birth >1 months, no vitamin D supplementation and calcium supplementation in the last 3 months, and daily dose of vitamin D supplementation ≤400 IU, time of sleeping <10 h every day, and outdoor activity <2 h every day (all *P*s < 0.05).

**TABLE 1:** General characteristics of vitamin D deficiency, the concentration of 25(OH)D, and obesity during the first 5 years of life: a cross‐sectional multicenter study (*n* = 5289)

Characteristics	*N*	Vitamin D deficiency		25(OH)D concentration		*N*	Obesity	*p*
		*N* (%)	*p*	*P*_50_ (*P*_25_–*P*_75_)	*p*		*N* (%)	
Gender								
Boy	2786	788 (28.3)	**.003**	65.1 (47.5–85.0)	**.002**	2783	177 (6.4)	**.000**
Girl	2503	803 (32.1)		62.7 (45.3–81.1)		2501	87 (3.5)	
Age								
0–11 months	960	221 (23.0)	**.000**	75.0 (52.4–97.9)	**<.001**	959	51 (5.3)	**.000**
12–23 months	912	194 (21.3)		70.0 (52.6–93.1)		910	24 (2.6)	
24–35 months	846	235 (27.8)		65.7 (47.0–81.4)		846	29 (3.4)	
36–47 months	884	286 (32.4)		61.0 (45.6–77.6)		884	39 (4.4)	
48–59 months	828	314 (37.9)		57.2 (43.7–72.7)		828	45 (5.4)	
60–71 months	859	341 (39.7)		55.5 (41.8–71.9)		857	76 (8.9)	
Age group								
0–23 months	1872	415 (22.2)	**<.001**	72.0 (52.5–96.1)	**<.001**	1869	75 (4.0)	**.015**
24–71 months	3417	1176 (34.4)		59.9 (44.1–76.5)		3415	189 (5.5)	
Total	5289	1591 (30.1)		64.0 (46.3–83.0)		5284	264 (5.0)	
Number of pregnancies								
1 Time	4040	1254 (31.0)	**.005**	63.3 (46.0–82.7)	**.042**	4037	192 (4.8)	.125
≥2 Times	1197	321 (26.8)		65.9 (49.0–84.0)		1195	70 (5.9)	
Parity								
1 Time	4125	1220 (29.6)	**.033**	64.0 (46.9–83.5)	**.009**	4121	174 (4.2)	.150
≥2 Times	761	196 (25.8)		67.0 (49.7–87.0)		761	41 (5.4)	
Gestational age								
Preterm infant	263	81 (30.8)	.887	64.7 (44.4–87.0)	.995	263	10 (3.8)	.450
Term infant	4858	1476 (30.4)		64.0 (46.0–83.0)		4853	234 (3.8)	
Delivery mode								
Spontaneous delivery	2241	692 (30.9)	.163	63.9 (45.6–83.7)	.302	2240	91 (4.1)	**.008**
Cesarean delivery	2936	854 (29.1)		64.2 (47.0–82.5)		2932	167 (5.7)	
Birth weight								
<2500 g	176	41 (23.3)	.133	70.8 (50.3–93.5)	**.006**	176	5 (2.8)	**.000**
2500–4000 g	4467	1354 (30.3)		64.0 (46.0–82.9)		4464	205 (4.6)	
≥4000 g	579	177 (30.6)		62.0 (47.0–79.4)		577	51 (8.8)	
Region of residence								
Urban	3738	1148 (30.7)	.124	63.0 (46.0–81.1)	**.001**	3733	195 (5.2)	.241
Rural	1550	443 (28.6)		65.8 (46.7–86.3)		1550	69 (4.5)	
Season of survey								
Spring	1554	580 (37.3)	**.000**	59.0 (41.0–76.5)	**.000**	1551	82 (5.3)	**.000**
Summer	2210	677 (30.6)		65.3 (44.4–89.0)		2209	74 (3.3)	
Autumn	672	139 (20.7)		69.0 (51.1–89.0)		672	23 (3.4)	
Winter	853	195 (22.9)		64.0 (50.9–79.0)		852	85 (10.0)	
Location in Jiangsu province								
Nanjing	989	180 (18.2)	**.000**	65.5 (54.0–81.0)	**.000**	987	42 (4.3)	.381
The Southern of Jiangsu	1277	668 (52.3)		48.0 (23.8–72.0)		1277	66 (5.2)	
Central Region of Jiangsu	952	301 (31.6)		62.9 (45.3–87.0)		952	42 (4.4)	
The Northern of Jiangsu	2071	442 (21.3)		70.0 (51.9–87.0)		2068	114 (5.5)	
Feeding patterns for infants from birth to 6 months								
Breast feeding	2702	828 (30.6)	.203	63.2 (46.0–82.0)	.362	2700	141 (5.2)	.578
Mixed feeding	1518	457 (30.1)		64.6 (46.4–81.6)		1515	74 (4.9)	
Artificial feeding	1023	283 (27.7)		65.0 (46.9–84.3)		1023	45 (4.4)	
Vitamin D supplementation for infants from birth to 6 months								
Yes	3726	1042 (28.0)	**.000**	65.8 (48.0–83.8)	**.000**	3721	158 (4.2)	**.005**
No	1346	471 (35.0)		60.0 (43.0–81.9)		1346	83 (6.2)	
The initial time of vitamin D supplementation after birth								
≤1 months	1641	420 (25.6)	**.014**	67.8 (49.5–87.7)	**.000**	1638	59 (3.6)	.107
>1 months	1944	569 (29.3)		64.0 (47.0–81.7)		1942	91 (4.7)	
The initial time of food supplementation								
≤4 months	306	102 (33.3)	.214	60.0 (43.7–82.2)	.0690	305	23 (7.5)	**.019**
>4 months	4545	1362 (30.0)		64.1 (46.4–82.8)		4541	208 (4.6)	
Vitamin D supplementation for children in the last 3 months								
Yes	1476	361 (24.5)	**.000**	70.0 (50.0, 93.6)	**.000**	1473	69 (4.7)	.587
No	3727	1202 (32.3)		61.1 (45.3, 79.0)		3725	188 (5.0)	
Daily dose of vitamin D supplementation for children in the last 3 months								
≤400 IU	898	239 (26.6)	**.023**	69.5 (47.7–91.6)	**.000**	897	40 (4.5)	.844
>400 IU	405	84 (20.7)		75.0 (54.6–99.9)		403	17 (4.2)	
Calcium supplementation for children in the last 3 months								
Yes	985	268 (27.2)	**.025**	69.0 (48.5–89.0)	**.000**	984	40 (4.1)	.191
No	4171	1287 (30.9)		63.0 (46.0–81.6)		4167	211 (5.1)	
Daily dose of calcium supplementation for children								
<300 mg	588	179 (30.4)	.073	67.1 (45.1–87.0)	.054	733	25 (3.4)	.305
≥300 mg	194	46 (23.7)		70.8 (51.0–91.1)		48	3 (6.3)	
Milk intake every day for children								
0–71 months								
<250 ml	2105	672 (31.9)	**.002**	60.4 (46.0–79.8)	**.000**	2133	98 (4.6)	.564
≥250 ml	2387	659 (27.6)		66.5 (48.4–85.2)		2358	100 (4.2)	
Meat intake every day for children								
0–71 months								
≤150.0 g	3830	1152 (30.1)	**.011**	63.4 (46.4–81.0)	**.000**	3826	179 (4.7)	**.002**
>150.0 g	197	76 (38.6)		54.0 (45.0–68.0)		197	19 (9.6)	
Egg intake every day for children								
0–71 months								
<60.0 g	3828	1140 (29.8)	.386	63.0 (46.5–81.0)	**.031**	3825	179 (4.7)	.195
≥60.0 g	392	125 (31.9)		69.0 (44.8–87.1)		390	24 (6.2)	
Time of sleeping every day for children								
<10 h	1569	558 (35.6)	**.000**	58.4 (43.0–77.3)	**.000**	1567	100 (6.4)	**.008**
10–12 h	2335	668 (28.6)		64.0 (47.3–82.0)		2334	101 (4.3)	
12–16 h	1316	343 (26.1)		71.0 (49.0–92.6)		1314	57 (4.3)	
>16 h	28	9 (32.1)		58.0 (42.9–75.1)		28	3 (10.7)	
Time of outdoor activity every day for children								
<2 h	2774	881 (31.8)	**.003**	62.6 (46.0–81.7)	**.000**	2770	145 (5.2)	.183
≥2 h	2370	663 (28.0)		66.0 (47.4–85.1)		2369	105 (4.4)	

*Note*: The bold values are statistically significant. In addition, the numbers in bold use the same statistical method. Mann Whitney U Nonparametric Test or K Independent Samples Median Nonparametric Test was used to describe median differences by continuous variables and chi‐square test was used to examine differences in categorical variables.

The association between obesity with vitamin D deficiency and the level of 25(OH)D in 0–71 months children, 0–23 months children, and 24–71 months children is shown in Table 2. The 25(OH)D levels in 0–71 months children with obesity were significantly lower than children with nonobesity (*P*_50_ = 59.8, 64.0 nmol/ml, respectively; *p* < .05); the similar results were observed in 24–71 months children; however, no significant difference was observed in 0–23 months children. The prevalence of vitamin D deficiency in 0–71 months children with obesity and nonobesity were 36.0% and 29.8% respectively, and there was significant difference; the similar results were observed in 24–71 months children; however, no significant difference was observed in 0–23 months children.

**TABLE 2:** The association between obesity with vitamin D deficiency and the level of 25(OH)D during the first 5 years of life, respectively

	Vitamin D deficiency			25(OH)D concentration	
	*N*	*N* (%)	*p*	*P*_50_ (*P*_25_ *–P*_75_)	*p*
ZBMI in 0–71 months children					
Obesity (ZBMI < *P*_95_)	5020	1495 (29.8)	**.032**	64.0 (46.5–83.0)	**.035**
Nonobesity (ZBMI ≥ *P*_95)_	264	95 (36.0)		59.8 (43.0–79.6)	
ZBMI in 0–23 months children					
Obesity (ZBMI < *P*_95_)	1777	401 (22.6)	.087	71.7 (52.0–96.0)	.145
Nonobesity (ZBMI ≥ *P*_95)_	93	14 (15.1)		78.3 (57.7–99.6)	
ZBMI in 24–71 months children					
Obesity (ZBMI < *P*_95_)	3245	1101 (34.0)	**.026**	60.0 (44.4–77.0)	**.043**
Nonobesity (ZBMI ≥ *P*_95)_	170	72 (42.4)		55.0 (40.5–71.5)	

*Note*: The bold values are statistically significant. In addition, the numbers in bold use the same statistical method. Mann Whitney U Nonparametric Test or K Independent Samples Median Nonparametric Test was used to describe median differences by continuous variables and chi‐square test was used to examine differences in categorical variables.

The unadjusted and adjusted associations of 25(OH)D with weight, ZWAZ, ZWHZ, and ZBMI in 0–71 months children, 0–23 months children, and 24–71 months children are shown in Table 3. In unadjusted model, for each unit increase in weight, ZWAZ and ZBMI, the level of 25(OH)D in 0–71 months children significantly decreased by 1.29 nmol/L, 1.27 nmol/L, and 1.12 nmol/L (β = −1.29, *p* < .01; β = −1.27, *p* < .01; β = −1.12, *p* < .01), respectively. The association between 25(OH)D with weight in 0–71 months children showed similarity in Models 1–4. The association between 25(OH)D with ZWAZ in 0–71 months children showed similarity in Model 1 and Model 4, whereas no relation appeared in Model 2 and Model 3. The association between 25(OH)D with ZBMI in 0–71 months children showed similarity in Model 1, Model 3, and Model 4, whereas no relation appeared in Model 2. The negative association between 25(OH)D with ZWHZ in 0–71 months children showed similarity in Model 1, whereas no relation appeared in unadjusted model, Model 2, Model 3, and Model 4. In unadjusted model, for each unit increase in weight, ZWAZ, ZBMI, and ZWHZ, the level of 25(OH)D in 24–71 months children significantly decreased by 0.85 nmol/L, 1.77 nmol/L, 1.42 nmol/L, and 1.20 nmol/L (β = −0.85, *p* < .01; β = −1.77, *p* < .01; β = −1.42, *p* < .01; β = −1.20, *p* < .01), respectively. The association between 25(OH)D with weight showed similarity in Models 1–4. The association between 25(OH)D with ZWAZ, ZWHZ, and ZBMI showed similarity in Model 1, Model 3, and Model 4, whereas no relation appeared in Model 2. However, in unadjusted model, no correlation between 25(OH)D with WAZ, ZWHZ, and ZBMI in 0–23 months was observed.

**TABLE 3:** The unadjusted and adjusted associations of 25(OH)D with weight, ZWAZ, ZWHZ, and ZBMI in children during the first 5 years of life (β coefficients and 95% confidence intervals)

Variable	β (95% CI)		
	0–71 months	0–23 months	24–71 months
Weight			
Unadjusted	−1.29 (−1.44, −1.13)**	−0.50 (−1.21, 0.22)	−0.85 (−1.07, −0.63)**
Model 1	−0.58 (−0.92, −0.24)**	−0.05 (−1.04, 0.93)	−0.63 (−0.98, −0.29)**
Model 2	−0.92 (−1.17, −0.67)**	−0.15 (−1.05, 0.75)	−0.37 (−0.69, −0.05)*
Model 3	−1.25 (−1.47, −1.02)**	−2.12 (−3.36, −0.89)*	−0.70 (−0.98, −0.42)**
Model 4	−1.31 (−1.51, −1.11)**	−0.92 (−1.70, −0.14)*	−0.82 (−1.09, −0.56)**
ZWAZ			
Unadjusted	−1.27 (−1.97, −0.56)**	−1.06 (−2.38, 0.26)	−1.77 (−2.56, −0.97)**
Model 1	−1.11 (−1.86, −0.36)**	−1.00 (−2.38, 0.39)	−1.43 (−2.31, −0.55)**
Model 2	−0.00 (−0.90, 0.90)	−0.22 (−1.73, 1.28)	−0.64 (−1.74, 0.46)
Model 3	−0.77 (−1.64, 0.11)	−0.02 (−1.94, 1.90)	−1.36 (−2.32, −0.40)**
Model 4	−0.84 (−1.62, −0.07)*	−0.73 (−2.11, 0.64)	−1.57 (−2.47, −0.66)**
ZWHZ			
Unadjusted	−0.56 (−1.29, 0.16)	−0.66 (−2.05, 0.72)	−1.20 (−2.00, −0.40)**
Model 1	−1.26 (−2.00, −0.51)**	−0.58 (−1.99, 0.83)	−1.64 (−2.49, −0.78)**
Model 2	0.14 (−0.79, 1.08)	0.32 (−1.27, 1.91)	−0.62 (−1.73, 0.49)
Model 3	−0.76 (−1.64, 0.11)	0.05 (−1.96, 2.07)	−1.40 (−2.34, −0.46)**
Model 4	−0.68 (−1.45, 0.09)	−0.27 (−1.68, 1.14)	−1.51 (−2.39, −0.64)**
ZBMI			
Unadjusted	−1.12 (−1.81, −0.44)**	−0.85 (−2.21, 0.51)	−1.42 (−2.16, −0.68)**
Model 1	−1.27 (−1.97, −0.56)**	−0.85 (−2.23, 0.53)	−1.54 (−2.33, −0.74)**
Model 2	−0.22 (−1.11, 0.66)	−0.02 (−1.57, 1.53)	−0.59 (−1.62, 0.44)
Model 3	−1.16 (−2.00, −0.34)**	−0.07 (−2.04, 1.90)	−1.53 (−2.41, −0.64)**
Model 4	−1.03 (−1.76, −0.30)**	−0.39 (−1.77, 1.00)	−1.62 (−2.44, −0.80)**

*Note*: Model 1: Sex, number of pregnancies, parity, birth weight, region of residence, season, and location in Jiangsu province. Model 2: Model 1 + vitamin D supplementation for infants from birth to 6 months, initial time of vitamin D supplementation after birth, and vitamin D and calcium supplementation for children in the last 3 months. Model 3: Model 1 + daily intake of milk, meat, and egg for children. Model 4: Model 1 + time of sleeping and outdoor activity every day for children.

** *p* < .01.

* *p* < .05.

Binary logistic regression and multiple linear regression for obesity and vitamin D deficiency and 25(OH)D concentrations in 0–71 months children, 24–71 months children, and 0–23 months children separately are presented in Table 4. Compared with 0–71 months children of ZBMI under the 95th percentile, 0–71 months children with obesity had higher risk of vitamin D deficiency (OR: 1.33; 95% CI: 1.02, 1.72, *p* < .05). The result showed similarity in Model 1, Model 3, and Model 4, whereas no relation appeared in Model 2. Compared with 24–71 months children of ZBMI under the 95th percentile, 24–71 months children with obesity had higher risk of vitamin D deficiency (OR: 1.42; 95% CI: 1.04, 1.95, *p* < .05). The result showed similarity in Model 1, Model 3, and Model 4, whereas no relation appeared in Model 2; moreover, no association was observed in 0–23 months. 25(OH)D in 0–71 months children with obesity was lower, 3.84 (β = −3.84, 95% CI = −7.58, −0.09, *p* < .05). The result showed similarity in Model 3, whereas no relation appeared in Model 1, Model 2, and Model 4. Furthermore, 25(OH)D in 24–71 months children with obesity was lower, 4.74 (β = −4.74, 95% CI = −8.98, −0.51, *p* < .05). The result showed similarity in Model 1 and Model 3, whereas no relation appeared in Model 2 and Model 4; however, no association was observed in 0–23 months.

**TABLE 4:** The unadjusted and adjusted associations between obesity with vitamin D deficiency and the level of 25(OH)D, respectively, during the first 5 years of life (odds ratios, β coefficients, and 95% confidence intervals)

	Vitamin D deficiency		Level of 25(OH)D	
	Obesity (ZBMI ≥ *P*_95_) OR (95% CI)	Reference group (ZBMI < *P*_95_)	Obesity (ZBMI ≥ *P*_95_) β (95% CI)	Reference group (ZBMI < *P*_95_)
0–71 months				
Unadjusted	1.33 (1.02, 1.72)*	Ref.	−3.84 (−7.58, −0.09)*	Ref.
Model 1	1.44 (1.08, 1.94)*	Ref.	−3.23 (−7.35, 0.90)	Ref.
Model 2	1.20 (0.82, 1.78)	Ref.	0.27 (−4.72, 5.26)	Ref.
Model 3	1.52 (1.06, 2.17)*	Ref.	−4.85 (−9.58, −0.11)*	Ref.
Model 4	1.43 (1.06, 1.92)*	Ref.	−2.70 (−6.85, 1.46)	Ref.
0–23 months				
Unadjusted	0.61 (0.34, 1.09)	Ref.	4.52 (−2.39, 11.43)	Ref.
Model 1	0.62 (0.34, 1.11)	Ref.	4.16 (−2.76, 11.09)	Ref.
Model 2	0.52 (0.24, 1.12)	Ref.	6.54 (−1.19, 14.27)	Ref.
Model 3	0.14 (0.02, 1.04)	Ref.	5.10 (−5.67, 15.88)	Ref.
Model 4	0.56 (0.30, 1.03)	Ref.	5.73 (−1.27, 12.74)	Ref.
24–71 months				
Unadjusted	1.42 (1.04, 1.95)*	Ref.	−4.74 (−8.98, −0.51)*	Ref.
Model 1	1.67 (1.15, 2.41)**	Ref.	−5.01 (−9.87, −0.16)*	Ref.
Model 2	1.48 (0.91, 2.41)	Ref.	−1.24 (−7.32, 4.84)	Ref.
Model 3	1.71 (1.14, 2.56)**	Ref.	−5.22 (−10.42, −0.02)*	Ref.
Model 4	1.68 (1.16, 2.44)**	Ref.	−4.83 (−9.74, 0.09)	Ref.

*Note*: Model 1: Sex, number of pregnancies, parity, birth weight, region of residence, season, and location in Jiangsu province. Model 2: Model 1 + vitamin D supplementation for infants from birth to 6 months, initial time of vitamin D supplementation after birth, and vitamin D and calcium supplementation for children in the last 3 months. Model 3: Model 1 + daily intake of milk, meat, and egg for children. Model 4: Model 1 + time of sleeping and outdoor activity every day for children.

** *p* < .01.

* *p* < .05.

## 4 DISCUSSION

During the first 5 years of life, the results showed that children with obesity had higher risk of vitamin D deficiency and the 25(OH)D was inversely related to weight, ZWAZ, and ZBMI in Mainland China. This relationship mainly depended on the results from the first 2–5 years of life, but not from the first 2 years of life. During the first 1000 days of life, that is, the age period from pregnancy to 24 months of birth, the maternal and infant nutrition and environment during this period can obviously affect children's health and future. So this study conducted a stratified analysis of age. Results for children aged 24–71 months were similar to those for children aged 0–71 months. The results showed 24–71 months children with obesity had higher risk of vitamin D deficiency and the 25(OH)D was inversely related to weight, ZWAZ, ZWHZ, and ZBMI in Mainland China. However, no association was observed in 0–23 months children. Due to the rapid growth from 0 to 23 months period, we speculate that this relationship from 0 to 71 months mainly depends on the results from 24 to 71 months. The conclusions of this study are novel.

The results in Table 1 showed that factors such as demographic, dietary intake, and physical activity are not only associated with obesity, but also with vitamin D deficiency. First, sex, number of pregnancies, parity, birth weight, region of residence, season, and location in Jiangsu province were adjusted in Model 1. But these factors did not affect the relationship between obesity and vitamin D deficiency in 0–71 months and 24–71 months children. Second, vitamin D supplementation for infants from birth to 6 months, the initial time of vitamin D supplementation after birth, and vitamin D and calcium supplementation for children in the last 3 months were adjusted based on Model 1. In 0–71 months and 24–71 months children, the relation between vitamin D levels and obesity were modified by vitamin D and calcium supplementation. These findings suggest the primacy of vitamin D and calcium supplementation as indicators of 25(OH)D in early childhood. Third, daily intake of milk, meat, and egg for children were adjusted based on Model 1. Although dietary factors did not affect the relationship between obesity and vitamin D deficiency in 0–71 months and 24–71 months children, those affect the relationship between ZWAZ, ZWHZ, and 25(OH)D in 0–71 months children. Finally, time of sleeping and outdoor activity every day for children were adjusted based on Model 1. Physical activity factors did not affect the relationship between obesity and vitamin D deficiency in 0–71 months and 24–71 months children.

ZWAZ is significantly negatively correlated with 25(OH)D in children aged 0–71 months and 24–71 months. The results showed similarity in the association between ZBMI and 25(OH)D. The negative association between ZWHZ and 25(OH)D was observed only in children aged 24–71 months. It can be seen that anthropometry indicators ZBMI is better than ZWHZ in the evaluation of childhood obesity in early life.

Nevertheless, several studies have attempted to establish a relation between vitamin D levels and obesity, but the results were controversial. There was no association of body composition with 25(OH)D of 79 children at 5 years of age in Dublin, Ireland (McVey et al., 2019). There was no significant relationship between BMI and vitamin D in 215 children who were 2–7 years old at Taleghani Pediatric Hospital, Iran (Mohammadian et al., 2014). There were no significant correlations between 25(OH)D and BMI (Spearman's ρ = −0.096, *p* = .389) in 83 very young obese children ages 2–6 years from the Chicago metropolitan (Creo et al., 2013). Surprisingly, a positive correlation was found between serum 25(OH)D and BMI *z* score (*p* < .05) in Swedish preschool age children (*n* = 90; mean age 54 ± 7.1 months) and fat‐free mass index (*p* = .014) in Danish children aged 4–8 years (*n* = 130; Mortensen et al., 2018; Ohlund et al., 2013). However, other study found it was a significant positive correlation between lean body mass, but not body fat and plasma 25(OH)D concentration (Foo et al., 2009). Therefore, body composition (lean mass and fat mass) and the distribution of fat mass (visceral vs. subcutaneous) should be measured in further research. In addition, it may be related to the small sample size of these studies.

The mechanisms behind the low 25(OH)D levels in obesity were not well understood. First, the storage, volumetric dilution, or sequestration of vitamin D in adipose tissue may lead to decreased bioavailability and best explain the low vitamin D status of obesity (Drincic et al., 2012; Greco et al., 2019). Second, the vitamin D exert effects on adipose tissue, influence adipose tissue development and function, and regulate lipolysis and adipogenesis process (adiponectin, leptin, resistin, peroxisome proliferator activator receptor [PPAR]‐γ), adipocyte differentiation, proliferation and apoptosis, the catabolic and anabolic activity of adipocytes, cytokine release and adipose tissue inflammation, oxidative stress, energy metabolism, and the expression of miRNAs (miR‐146a, miR‐150, and miR‐155) in adipose tissue (Abbas, 2017; Ruiz‐Ojeda et al., 2018). Third, vitamin D has a modulatory effect on pancreas, and the beta cells in particular, modulates insulin synthesis and secretion, alters insulin sensitivity by stimulating the expression of insulin sensitivity genes, by activating peroxisome proliferator‐activated receptor delta, and by increasing activity of the insulin receptor gene and the total number of insulin receptors (Calle et al., 2008; Dunlop et al., 2005; Maestro et al., 2003). Fourth, it has an essential role in modulating immune response and inflammation in adipose tissue and adipocytes, inhibits the proinflammatory cytokines tumor necrosis factor (TNF‐alpha), IL‐1b, IL‐6, IL‐8, IL‐12, regulates the differentiation, infiltration, and transformation of macrophages, blocks the differentiation of dendritic cells, and inhibits lymphocyte proliferation (Abbas, 2017; Chang & Kim, 2017; Mehmood & Papandreou, 2016). Finally, VDR (vitamin D receptor) are widely distributed along several body tissues, their gene polymorphisms may affect the risk of vitamin D‐related metabolic disorders, and could adjust the receptor effectiveness according to vitamin D status (Hasan et al., 2017; María et al., 2018). Further epidemiological and also basic molecular studies in animal models and human biopsies would help to address those issues.

The strengths of our study are as follows. First, data of the association between vitamin D deficiencies with obesity worldwide focusing on the early life cycle from birth to 5 years are lacking, to the best of our knowledge. Second, this was large multicenter cross‐sectional study. Finally, the study had considered the potential confounding variables including demographic characteristics, dietary intake, vitamin D and calcium supplementation, and physical activity in children.

Nevertheless, the limitations were worth mentioning. First, we cannot infer causality between 25(OH)D and obesity due to cross‐sectional design of the study. Second, the various forms of vitamin D such as 25(OH)D_3_ and 25(OH)D_2_ were unable to be distinguished. Finally, in this study, we used the children's BMI as the indicator of anthropometric status, and future studies should observe the association between body composition (lean mass and fat mass) and the type of the distribution of fat mass (visceral vs. subcutaneous) and 25(OH)D levels using more precise measures of adiposity.

## 5 CONCLUSION

Vitamin D deficiency was observed in children with greater adiposity during the first 5 years of life. However, the results mainly came from those in the age group of 2–5 years instead of the first 2 years in their lives.

## AUTHOR CONTRIBUTIONS

RQ and YZ designed the study. HH, HL, KY, YW, WZ, HQ, YN, LZ, GY, GL, and AW collected the data. YZ conducted the statistical analysis and interpretation and drafted the manuscript. RQ revised the manuscript. All authors were involved in the writing of the manuscript and had final approval of the submitted and published versions.

## FUNDING INFORMATION

This work was funded by the construction fund for Young Scholars Fostering Fund of the First Affiliated Hospital of Nanjing Medical University (PY2021050) and Key subjects of women and children of Jiangsu province (FXK201203).

## CONFLICT OF INTEREST

The authors declare that they have no known competing financial interests or personal relationships that could have appeared to influence the study reported in this article.

## ETHICAL APPROVAL

The study protocol was approved by the institutional review board of the First Affiliated Hospital of Nanjing Medical University (2014‐SR‐167).

## Data Availability

Data are available on request from the corresponding author.

## References

[fsn33145-bib-0001] Abbas, M. A. (2017). Physiological functions of vitamin D in adipose tissue. The Journal of Steroid Biochemistry and Molecular Biology, Pt B(165), 369–381. 10.1016/j.jsbmb.2016.08.004 27520301

[fsn33145-bib-0002] Avagyan, D. , Neupane, S. P. , Gundersen, T. E. , & Madar, A. A. (2016). Vitamin D status in pre‐school children in rural Nepal. Public Health Nutrition, 19(3), 470–476. 25827017 10.1017/S136898001500083X PMC10271105

[fsn33145-bib-0003] Calle, C. , Maestro, B. , & García‐Arencibia, M. (2008). Genomic actions of 1,25‐dihydroxyvitamin D3 on insulin receptor gene expression, insulin receptor number and insulin activity in the kidney, liver and adipose tissue of streptozotocin‐induced diabetic rats. BMC Molecular Biology, 9, 65 https://www.ncbi.nlm.nih.gov/pmc/articles/PMC2533347/pdf/1471‐2199‐9‐65.pdf 18638371 10.1186/1471-2199-9-65 PMC2533347

[fsn33145-bib-0004] Capital Institute of Pediatrics The Coordinating Study Group of Nine Cities on the Physical Growth and Development of Children . (2018). A national epidemiological survey on obesity of children under seven years of age in nine cities of China in 2016. Chinese Journal of Pediatrics, 56(10), 745–752. 10.3760/cma.j.issn.0578-1310.2018.10.006 30293278

[fsn33145-bib-0005] Chang, E. , & Kim, Y. (2017). Vitamin D insufficiency exacerbates adipose tissue macrophage infiltration and decreases AMPK/SIRT1 activity in obese rats. Nutrients, 9(4), 338. 28353634 10.3390/nu9040338 PMC5409677

[fsn33145-bib-0006] Corica, D. , Zusi, C. , Olivieri, F. , Marigliano, M. , Piona, C. , Fornari, E. , Morandi, A. , Corradi, M. , Miraglia Del Giudice, E. , Gatti, D. , Rossini, M. , Bonadonna, R. C. , & Maffeis, C. (2019). Vitamin D affects insulin sensitivity and β‐cell function in obese non‐diabetic youths. European Journal of Endocrinology, 181(4), 439–450. 31408845 10.1530/EJE-19-0369

[fsn33145-bib-0007] Creo, A. L. , Rosen, J. S. , Ariza, A. J. , Hidaka, K. M. , & Binns, H. J. (2013). Vitamin D levels, insulin resistance, and cardiovascular risks in very young obese children. Journal of Pediatric Endocrinology & Metabolism, 26(1–2), 97–104. 23382301 10.1515/jpem-2012-0244

[fsn33145-bib-0008] Drincic, A. T. , Armas, L. A. , Van Diest, E. E. , & Heaney, R. P. (2012). Volumetric dilution, rather than sequestration best explains the low vitamin D status of obesity. Obesity (Silver Spring), 20(7), 1444–1448. 10.1038/oby.2011.404 22262154

[fsn33145-bib-0009] Dunlop, T. W. , Väisänen, S. , Frank, C. , Molnár, F. , Sinkkonen, L. , & Carlberg, C. (2005). The human peroxisome proliferator‐activated receptor delta gene is a primary target of 1alpha,25‐dihydroxyvitamin D3 and its nuclear receptor. Journal of Molecular Biology, 349(2), 248–260 https://www.sciencedirect.com/science/article/abs/pii/S0022283605003463?via%3Dihub 15890193 10.1016/j.jmb.2005.03.060

[fsn33145-bib-0010] Foo, L. H. , Zhang, Q. , Zhu, K. , Ma, G. , Trube, A. , Greenfield, H. , & Fraser, D. R. (2009). Relationship between vitamin D status, body composition and physical exercise of adolescent girls in Beijing. Osteoporosis International, 20(3), 417–425. 18629568 10.1007/s00198-008-0667-2

[fsn33145-bib-0011] Fu, Y. , Hu, Y. , Qin, Z. , Zhao, Y. , Yang, Z. , Li, Y. , Liang, G. , Lv, H. , Hong, H. , Song, Y. , Wei, Y. , Yue, H. , Zheng, W. , Liu, G. , Ni, Y. , Zhu, M. , Wu, A. , Yan, J. , Ji, C. , … Qin, R. (2016). Association of serum 25‐hydroxyvitamin D status with bone mineral density in 0‐7 year old children. Oncotarget, 7(49), 80811–80819. 27821808 10.18632/oncotarget.13097 PMC5348357

[fsn33145-bib-0012] Greco, E. A. , Lenzi, A. , & Migliaccio, S. (2019). Role of hypovitaminosis D in the pathogenesis of obesity‐induced insulin resistance. Nutrients, 11(7), 1507. 31266190 10.3390/nu11071506 PMC6682882

[fsn33145-bib-0013] Hasan, H. A. , AbuOdeh, R. O. , Muda, W. A. , Mohamed, H. J. , & Samsudin, A. R. (2017). Association of vitamin D receptor gene polymorphisms with metabolic syndrome and its components among adult Arabs from The United Arab Emirates. Diabetes and Metabolic Syndrome: Clinical Research and Reviews, 11(Suppl 2), S531–S537. 10.1016/j.dsx.2017.03.047 28392355

[fsn33145-bib-0014] Holick, M. F. , Binkley, N. C. , Bischoff‐Ferrari, H. A. , Gordon, C. M. , Hanley, D. A. , Heaney, R. P. , Murad, M. H. , & Weaver, C. M. (2011). Evaluation, treatment, and prevention of vitamin D deficiency: An Endocrine Society clinical practice guideline. The Journal of Clinical Endocrinology & Metabolism, 96(7), 1911–1930. 10.1210/jc.2011-0385 21646368

[fsn33145-bib-0015] Jaksic, M. , Martinovic, M. , Gligorovic‐Barhanovic, N. , Vujacic, A. , Djurovic, D. , & Nedovic‐Vukovic, M. (2019). Association between inflammation, oxidative stress, vitamin D, copper and zinc with pre‐obesity and obesity in school children from the city of Podgorica, Montenegro. Journal of Pediatric Endocrinology & Metabolism, 32(9), 951–957. 31444965 10.1515/jpem-2019-0086

[fsn33145-bib-0016] Karin, Z. , Gilic, B. , Supe Domic, D. , Sarac, Z. , Ercegovic, K. , Zenic, N. , Uljevic, O. , Peric, M. , & Markic, J. (2018). Vitamin D status and analysis of specific correlates in preschool children: A cross‐sectional study in southern Croatia. International Journal of Environmental Research and Public Health, 15(11), 2503–2517. 10.3390/ijerph15112503 30413103 PMC6266977

[fsn33145-bib-0017] Kassab, M. , Shaban, I. , Mohammad, K. , & Creedy, D. K. (2016). Prevalence of hypovitaminosis D among Jordanian healthy infants: A descriptive cross sectional study. Journal of Pediatric Nursing, 31(2), e119–e125. 10.1016/j.pedn.2015.10.004 26577996

[fsn33145-bib-0018] Lee, E. Y. , Spence, J. C. , & Carson, V. (2017). Television viewing, reading, physical activity and brain development among young south Korean children. Journal of Science and Medicine in Sport, 20(7), 672–677. 10.1016/j.jsams.2016.11.014 28169149

[fsn33145-bib-0019] Lu, Q. , Tao, F. , Hou, F. , Zhang, Z. , & Ren, L. L. (2016). Emotion regulation, emotional eating and the energy‐rich dietary pattern. A population‐based study in Chinese adolescents. Appetite, 99, 149–156. 10.1016/j.appet.2016.01.011 26792769

[fsn33145-bib-0020] Maestro, B. , Dávila, N. , Carranza, M. C. , & Calle, C. (2003). Identification of a vitamin D response element in the human insulin receptor gene promoter. The Journal of Steroid Biochemistry and Molecular Biology, 84(2–3), 223–230 https://www.sciencedirect.com/science/article/abs/pii/S0960076003000323?via%3Dihub 12711007 10.1016/s0960-0760(03)00032-3

[fsn33145-bib-0021] María, C. R. , Antonio, C. J. , Jacqueline, S. R. , Emilio, G. J. , Sofia, V. , Javier, M. , & Blanca, R. M. (2018). Genetic association analysis of vitamin D receptor gene polymorphisms and obesity‐related phenotypes. Gene, 640, 51–56. 29032145 10.1016/j.gene.2017.10.029

[fsn33145-bib-0022] McVey, M. K. , Geraghty, A. A. , O'Brien, E. C. , Kilbane, M. T. , Crowley, R. K. , Twomey, P. J. , McKenna, M. J. , & McAuliffe, F. M. (2019). An exploratory analysis of associations of diet, sun exposure, and body composition with 25OHD at five years of age: Findings from the ROLO kids study. The Journal of Steroid Biochemistry and Molecular Biology, 188, 111–116. 30605775 10.1016/j.jsbmb.2018.12.014

[fsn33145-bib-0023] Mehmood, Z. H. , & Papandreou, D. (2016). An updated mini review of vitamin D and obesity: Adipogenesis and inflammation state. Open Access Macedonian Journal of Medical Sciences, 4(3), 526–532 https://www.ncbi.nlm.nih.gov/pmc/articles/PMC5042647/ 27703587 10.3889/oamjms.2016.103 PMC5042647

[fsn33145-bib-0024] Mohammadian, S. , Mortezazadeh, R. , Zaeri, H. , & Vakili, M. A. (2014). Relationship between 25‐hydroxy vitamin‐D and obesity in 2‐7 years old children referred to a Paediatric Hospital in Iran. Journal of Clinical and Diagnostic Research, 8(9), Pc06‐08. 10.7860/jcdr/2014/8282.4810 PMC4225948 25386496

[fsn33145-bib-0025] Mortensen, C. , Mølgaard, C. , Hauger, H. , Kristensen, M. , & Damsgaard, C. T. (2018). Sun behaviour and physical activity associated with autumn vitamin D status in 4‐8‐year‐old Danish children. Public Health Nutrition, 21(17), 3158–3167. 10.1017/s1368980018002094 30189911 PMC10261082

[fsn33145-bib-0026] Moschonis, G. , Androutsos, O. , Hulshof, T. , Dracopoulou, M. , Chrousos, G. P. , & Manios, Y. (2018). Vitamin D insufficiency is associated with insulin resistance independently of obesity in primary schoolchildren. The healthy growth study. Pediatric Diabetes, 19(5), 866–873. 10.1111/pedi.12678 29608042

[fsn33145-bib-0027] Ogden, C. L. , Carroll, M. D. , Kit, B. K. , & Flegal, K. M. (2014). Prevalence of childhood and adult obesity in the United States, 2011‐2012. JAMA, 311(8), 806–814 https://jamanetwork.com/journals/jama/articlepdf/1832542/joi140013.pdf 24570244 10.1001/jama.2014.732 PMC4770258

[fsn33145-bib-0028] Ohlund, I. , Silfverdal, S. A. , Hernell, O. , & Lind, T. (2013). Serum 25‐hydroxyvitamin d levels in preschool‐age children in northern Sweden are inadequate after summer and diminish further during winter. Journal of Pediatric Gastroenterology and Nutrition, 56(5), 551–555. 23274340 10.1097/MPG.0b013e3182838e5b

[fsn33145-bib-0029] Plesner, J. L. , Dahl, M. , Fonvig, C. E. , Nielsen, T. R. H. , Kloppenborg, J. T. , Pedersen, O. , Hansen, T. , & Holm, J. C. (2018). Obesity is associated with vitamin D deficiency in Danish children and adolescents. Journal of Pediatric Endocrinology & Metabolism, 1(31), 53–61. 10.1515/jpem-2017-0246 29197860

[fsn33145-bib-0030] Reinehr, T. , Langrock, C. , Hamelmann, E. , Lücke, T. , Koerner‐Rettberg, C. , Holtmann, M. , Legenbauer, T. , Gest, S. , Frank, M. , Schmidt, B. , Radkowski, K. , & Jöckel, K. H. (2018). 25‐Hydroxvitamin D concentrations are not lower in children with bronchial asthma, atopic dermatitis, obesity, or attention‐deficient/hyperactivity disorder than in healthy children. Nutrition Research, 52, 39–47 https://www.sciencedirect.com/science/article/pii/S0271531717309466?via%3Dihub 29764626 10.1016/j.nutres.2018.01.002

[fsn33145-bib-0031] Ruiz‐Ojeda, F. J. , Anguita‐Ruiz, A. , Leis, R. , & Aguilera, C. M. (2018). Genetic factors and molecular mechanisms of vitamin D and obesity relationship. Annals of Nutrition & Metabolism, 73(2), 89–99. 29982250 10.1159/000490669

[fsn33145-bib-0032] Sempos, C. T. , Betz, J. M. , Camara, J. E. , Carter, G. D. , Cavalier, E. , Clarke, M. W. , Dowling, K. D. , Durazo‐Arvizu, R. , Hoofnagle, A. N. , Liu, A. , Sarafin, K. , Wise, S. A. , & Coates, P. M. (2017). General steps to standardize the laboratory measurement of serum total 25‐hydroxyvitamin D. Journal of AOAC International, 100(5), 1230–1233. 10.5740/jaoacint.17-0259 28766476

[fsn33145-bib-0033] WHO Multicentre Growth Reference Study Group . (2006). WHO child growth standards: Methods and development: Length/height‐for‐age, weight‐for‐age, weight‐for‐length, weight‐for‐height and body mass index‐for‐age. World Health Organization.

[fsn33145-bib-0034] Zhao, Y. , Qin, R. , Ma, X. , Qin, Z. , Yang, Z. , Hong, H. , Lv, H. , Ye, K. , Wei, Y. , Zheng, W. , Qi, H. , Ni, Y. , Zhang, L. , Yan, J. , Liu, G. , & Wu, A. (2020). Adiposity is not beneficial to bone mineral density in 0‐5 year old Chinese children: The Jiangsu bone health study. Obesity Research & Clinical Practice, 14(1), 39–46. 31879074 10.1016/j.orcp.2019.10.011

